# Tomato in the spotlight: light regulation of whole-plant physiology

**DOI:** 10.1093/jxb/eraf315

**Published:** 2025-07-15

**Authors:** Ep Heuvelink, Liana G Acevedo-Siaca, Bram Van de Poel, Laura Van der Jeucht, Silvere Vialet-Chabrand, Kathy Steppe, Yongran Ji, Oliver Körner, Paul Kusuma, Silvia Langer, Tao Li, Wim Van Ieperen, Julian C Verdonk, Ana Cristina Zepeda, Yuqi Zhang, Leo F M Marcelis

**Affiliations:** Horticulture and Product Physiology, Wageningen University, Droevendaalsesteeg 1, 6708 PB Wageningen, Netherlands; Horticulture and Product Physiology, Wageningen University, Droevendaalsesteeg 1, 6708 PB Wageningen, Netherlands; Division of Crop Biotechnics, Department of Biosystems, University of Leuven, Willem de Croylaan 42, 3001 Leuven, Belgium; KU Leuven Plant Institute, University of Leuven, Kasteelpark Arenberg 31, 3001 Leuven, Belgium; Division of Crop Biotechnics, Department of Biosystems, University of Leuven, Willem de Croylaan 42, 3001 Leuven, Belgium; KU Leuven Plant Institute, University of Leuven, Kasteelpark Arenberg 31, 3001 Leuven, Belgium; Horticulture and Product Physiology, Wageningen University, Droevendaalsesteeg 1, 6708 PB Wageningen, Netherlands; Laboratory of Plant Ecology, Faculty of Bioscience Engineering, Ghent University, Coupure links 653, B-9000 Ghent, Belgium; Horticulture and Product Physiology, Wageningen University, Droevendaalsesteeg 1, 6708 PB Wageningen, Netherlands; Leibniz-Institute of Vegetable and Ornamental Crops (IGZ), Theodor-Echtermeyer-Weg 1, 14979 Großbeeren, Germany; Horticulture and Product Physiology, Wageningen University, Droevendaalsesteeg 1, 6708 PB Wageningen, Netherlands; Horticulture and Product Physiology, Wageningen University, Droevendaalsesteeg 1, 6708 PB Wageningen, Netherlands; Institute of Environment and Sustainable Development in Agriculture, Chinese Academy of Agricultural Sciences, Beijing 100081, China; Horticulture and Product Physiology, Wageningen University, Droevendaalsesteeg 1, 6708 PB Wageningen, Netherlands; Horticulture and Product Physiology, Wageningen University, Droevendaalsesteeg 1, 6708 PB Wageningen, Netherlands; Horticulture and Product Physiology, Wageningen University, Droevendaalsesteeg 1, 6708 PB Wageningen, Netherlands; Institute of Environment and Sustainable Development in Agriculture, Chinese Academy of Agricultural Sciences, Beijing 100081, China; Horticulture and Product Physiology, Wageningen University, Droevendaalsesteeg 1, 6708 PB Wageningen, Netherlands; INRAE-Bordeaux, France

**Keywords:** Assimilate partitioning, cryptochrome, fruit quality, morphology, photobiology, photosynthesis, phytochrome, plant–water relations, *Solanum lycopersicum*, tomato, transpiration

## Abstract

The introduction of light-emitting diodes in plant research and controlled-environment agriculture has given a boost to understanding how light regulates physiology. Here, we review the regulation of whole-plant physiological processes by light in tomato (*Solanum lycopersicum*), with emphasis on morphogenesis, light interception, photosynthesis, source–sink interactions, assimilate partitioning, fruit set, fruit development, and plant–water relations and how this controls plant growth and fruit quality. Five key aspects of light determine the ultimate plant response, namely intensity, photoperiod, spectrum, directionality, and energy. Tomato possesses five phytochromes, four cryptochromes, two phototropins, one zeitlupe, and one UV-B photoreceptor. Via spectral sensing and photosynthesis, light affects plant morphology, which in turn affects the light interception and consequently whole-plant carbon assimilation. Photosynthesis and carbon partitioning are dynamic processes affected by light. Furthermore, light plays a pivotal role in regulating plant–water–nutrient dynamics by influencing transpiration, stomatal conductance, hydraulic conductance, and cell-wall properties. Changes in light intensity and spectrum can also increase contents of ascorbate, carotenoids, sugars, and volatiles, thereby improving fruit quality. The complex physiological responses of tomato plants to the five aspects of light and their interactions create effectively endless opportunities for future scientific research aimed at improving light-use efficiency, yield, and quality.

## Teaser

We review five key aspects of light (intensity, photoperiod, spectrum, directionality, and energy) that regulate whole-plant physiological processes including morphogenesis, photosynthesis, source–sink interactions, assimilate partitioning, fruit development, and plant–water relations.

## Introduction

**Fig. 1.**
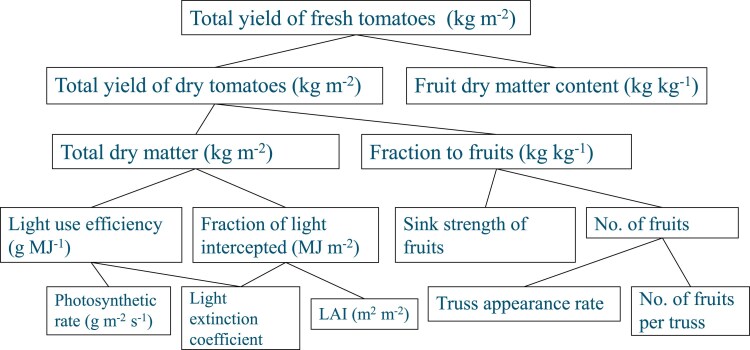
Yield component analysis. The fresh fruit yield of a tomato crop can be expressed as the product of total crop dry matter production and the fraction of dry matter partitioned to the fruits (harvest index), divided by the fruit dry matter content. The latter parameter determines how much fruit fresh mass (yield) results from the dry matter partitioned into the fruit. Dry matter production is primarily determined by crop photosynthesis, while photosynthesis to a large extent depends on light interception, which differs with leaf area index (LAI) and canopy light-extinction coefficient. Light-use efficiency is the ratio between dry matter production and the amount of intercepted light. High dry matter production only results in a high yield when a large fraction of assimilates is partitioned to the fruits. Partitioning to the fruits depends on the number of fruit (fruit set) and the capacity to import assimilates (sink strength) of individual fruits.

Tomato (*Solanum lycopersicum*) is the second most important vegetable crop after potato, it is the most valuable fruit crop globally (Heuvelink *et al*., 2024), and it is the prominent model system for scientific research. Over the past 40 years fruit yield in greenhouses has increased by more than 200% in the Netherlands, as a result of higher light transmissivity of the greenhouse cover (with a 1% light increment resulting in a 0.7–1% increase in yield; Marcelis *et al*., 2006), use of supplementary lighting, advanced greenhouse technologies and cultivation techniques, and improved climate control and genetic improvement (Heuvelink *et al*., 2024). Such increases can be investigated using yield component analyses, which provide a powerful tool for elucidating the underlying reasons for differences in yield (Fig. 1). For example, Higashide and Heuvelink (2009) examined eight greenhouse cultivars introduced between 1950 and 2003 and concluded that modern cultivars have much higher yields than older ones because of improved light-use efficiency resulting from higher leaf photosynthesis rates and improved plant architecture resulting in lower light-extinction coefficients, which allow light to penetrate deeper in the canopy. Heuvelink *et al*. (Supplementary Fig. S1 S1) conducted a similar study with six cultivars introduced between 1975 and 2013. Combining both sets of results, yield has improved on average by 0.7% each year due to better cultivars, totally ∼28% over 40 years. This suggests that breeding has contributed ∼15% to the total yield improvement in tomato (i.e. 30% out of 200%).

**Fig. 2.**
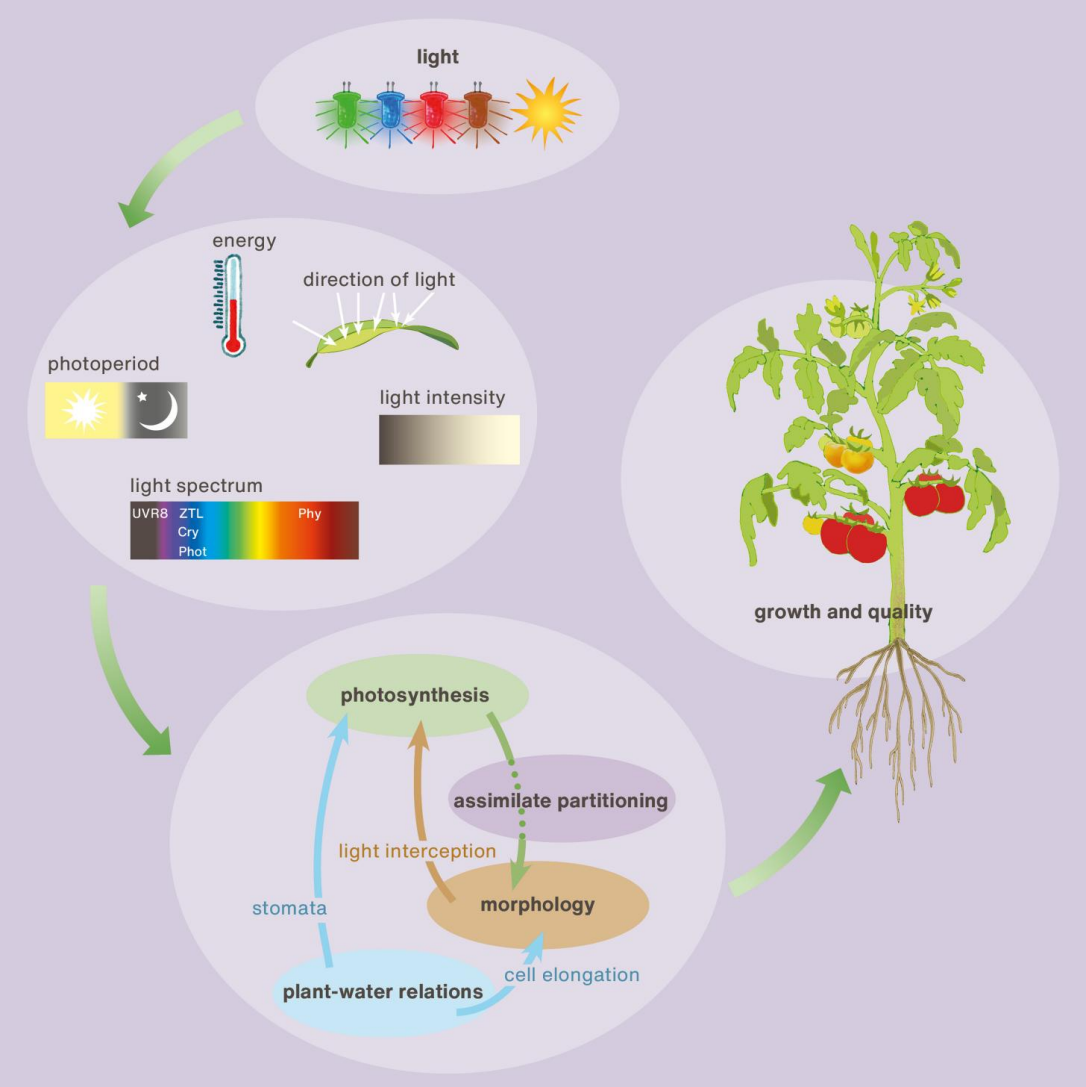
Schematic representation of the five key aspects of light that regulate whole-plant physiological processes: intensity, photoperiod, spectrum, directionality, and energy (heat). All five affect processes such as photosynthesis, morphology, assimilate partitioning, plant–water relations, and their interrelationships. All these processes ultimately affect plant growth and quality of the fruits. UVR8, ZTL, Cry, Phot, and Phy are photoreceptors acting in different regions of the spectrum (see Fig. 3).

The introduction of light-emitting diodes (LEDs) in controlled-environment agriculture as well as the increase in vertical farming has boosted research on light regulation of plant functioning (Pattison *et al*., 2018; Lazzarin *et al*., 2021; van Delden *et al*., 2021). Light has a number of different characteristics that all regulate numerous physiological and morphological processes, with the five main ones being intensity, photoperiod, spectrum, directionality, and energy (Fig. 2). All these characteristics can affect photosynthesis and morphology, and subsequently light can affect partitioning of the carbon assimilates in the plant, which determines the growth of the different organs. Photosynthesis of a crop strongly depends on the amount of intercepted light, which directly depends on intensity and spectrum, and indirectly depends on the effects of light on plant morphology. Light also strongly affects plant–water relations, which has consequences for photosynthesis (e.g. via stomatal regulation) as well as for morphology (e.g. via cell elongation). Collectively, these processes influence plant growth, and fruit yield and quality (Fig. 2).

Here, we aim to review the regulation of whole-plant physiological processes in tomato by LEDs and solar light, with emphasis on morphogenesis, light interception, photosynthesis, source–sink interactions, assimilate partitioning, fruit set, fruit development, and plant–water relations and how this controls plant growth and fruit quality.

## Tomato photoreceptors and their main functions

**Fig. 3.**
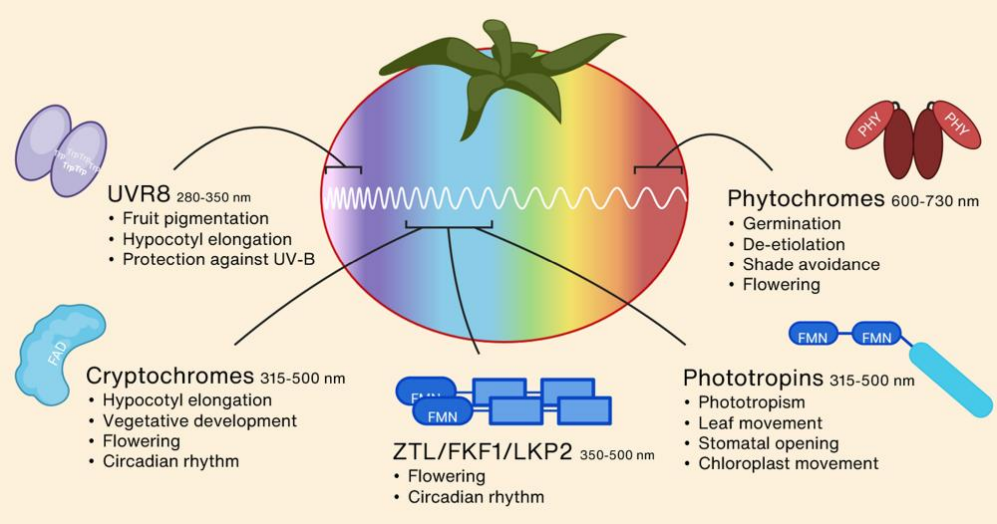
Schematic representation of the different photoreceptors in tomato, their respective absorption spectra, corresponding chromophores, and main functions. FAD, flavin adenine dinucleotide; FMN, flavin mononucleotide; PHY, phytochromobilin; Trp, tryptophan residues.

Plants rely on photoreceptors to sense spectral changes and initiate various physiological responses. Most plants, including tomato, possess five types of specialized photoreceptors (Fig. 3). Phytochromes, which mostly detect red and far-red (FR) light, have been well-studied since their discovery in 1959 by scientists at the US Department for Agriculture (Butler *et al*., 1959). In the 1990s, two types of blue-light photoreceptors were discovered, namely cryptochromes (Ahmad and Cashmore, 1993) and phototropins (Huala *et al*., 1997), and more recently the ZTL/FKF1/LKP2 family of blue-light photoreceptors (Somers *et al*., 2000) and the UV-B receptor UVR8 (Kliebenstein *et al*., 2002) were also discovered. The combined functioning and cross-signalling of these photoreceptors regulate growth, development, and adaptation to environmental changes. An overview of the known photoreceptor mutants and transgenic lines, and their major functions in tomato are provided in Supplementary Table S1.

### Phytochromes

Phytochromes are molecular switches that shift between their active and inactive states via a photosensitive tetrapyrrole bilin chromophore (phytochromobilin) that is covalently bound to a conserved cysteine residue. The phytochrome structure is highly conserved across plants and bacteria, with an N-terminal photosensory region including three domains [Pern-Arnt-Sim (PAS), cGMP phosphodiesterase, Adenylate cyclase, FhlA (GAF), and phytochrome-specific (PHY)] and a C-terminal histidine kinase domain (Rockwell *et al*., 2006). Phytochromes can be categorized in two classes (Tokuhisa *et al*., 1985; Lopéz-Juez *et al*., 1990). Class I are light-labile, activated by FR, and are important under low-light conditions (very low fluence response in the range 0.1–10 μmol m^−2^ s^−1^, and low fluence response in the range 10–100 μmol m^−2^ s^−1^) (Abe *et al*., 1985). They play a role in seedling germination and de-etiolation. Class II are light-stable, activated by red, and are important in long exposure to high light conditions: (high irradiance response >100 μmol m^–2^ s^−1^ (Abe *et al*., 1985). They play a role in different processes including elongation and flowering.

Tomato possesses five phytochromes: phyA (class I), phyB1, phyB2, phyE, and phyF (all class II). The duplication of phyB into phyB1 and phyB2 occurred independently in the *Solanaceae* (Hauser *et al*., 1995). PhyB1 and phyB2 have specific (e.g. phyB1 regulates gravitropism and phototropism) and overlapping (e.g. regulation of de-etiolation) functions in tomato (Carlson *et al*., 2020). PhyF belongs to a novel sub-family in tomato (Hauser *et al*., 1995).

Phytochromes are essential in the shade-avoidance syndrome of plants, which is mainly facilitated by phyB1, phyB2, and phyE in tomato (Schrager-Lavelle *et al*., 2016). A low red:FR ratio leads to a series of architectural changes (see below). PhyA has been shown to protect tomato plants against continuous light stress (Velez-Ramirez *et al*., 2019). Recently, phyF was shown to control de-etiolation together with phyB1/B2 and root development together with phyA in tomato (Balderrama *et al*., 2023). Phytochromes also control tomato fruit ripening (Gupta *et al*., 2014), and Bianchetti *et al*. (2020) have shown that phyA, phyB1, and phyB2 can also act as temperature sensors in tomato.

### Cryptochromes

Cryptochromes are a class of photoreceptors that sense blue light and UV-A (315–400 nm; Ahmad and Cashmore, 1993; Fantini *et al*., 2019) using an associated flavin adenine dinucleotide chromophore (Lin and Todo, 2005). Cryptochromes are inactive monomers in the dark but form active homo-oligomers after photon excitation, which can be dark-inactivated (Más *et al*., 2000; Sang *et al*., 2005; Q. Liu *et al*., 2020). Cryptochromes activate a photo-responsive pathway by interacting with other downstream signalling components (Sang *et al*., 2005; Liu *et al*., 2008). Whereas most plants that have two cryptochromes (cry1 and cry2), tomato has four, namely CRY1A, CRY1B, CRY2, and CRY3 (Perrotta *et al*., 2000), which regulate blue-light-dependent hypocotyl elongation of seedlings (Ninu *et al*., 1999), vegetative development (e.g. internode elongation, root development), and flowering (Fantini *et al*., 2019). Cryptochromes also take part in the synthesis of pigments such as anthocyanins, carotenoids, and chlorophyll in both leaves and fruit (Ninu *et al*., 1999; Fantini *et al*., 2019). Overexpression of *CRY2* in tomato confirms its regulatory role in vegetative development, flowering time, and fruit antioxidant content (Giliberto *et al*., 2005). Cryptochromes also regulate circadian biology (Lopez *et al*., 2012), including leaf movements (Fantini *et al*., 2019). Under nutrient starvation, cryptochromes can modulate uptake in tomato (D’Amico-Damião *et al*., 2022).

### Phototropins

Phototropins are blue-light receptors that regulate not only phototropism, but also chloroplast movement, stomatal opening, and leaf movement (Briggs and Christie, 2002). Tomato possesses two phototropins, PHOT1 and PHOT2 (Sharma *et al*., 2014), which act redundantly in regulating these processes, although PHOT1 is more sensitive (<1 μmol m^−2^ s^−1^) whilst PHOT2 seems to be only active in higher light conditions (>1 μmol m^−2^ s^−1^) (Sakai *et al*., 2001; Briggs and Christie, 2002; Christie, 2007). Both contain conserved light, oxygen, voltage 1 (LOV1) and LOV2 domains, with a Hinge1 region in between, and an N-terminal kinase domain, the latter being involved in phototropin signalling. These LOV domains associate with a flavin mononucleotide (FMN) chromophore and undergo a structural change when illuminated with blue light (Briggs and Christie, 2002).

Although phototropins in tomato have been poorly studied, findings of Sharma *et al*. (2014) suggest that, in concert with phytochromes, phot1 is involved in de-etiolation and hypocotyl orientation during the early stages of photomorphogenesis of tomato seedlings. Phototropins can also regulate the carotenoid content of tomato fruit (Kilambi *et al*., 2021).

### The ZEITLUPE/FLAVIN BINDING, KELCH REPEAT, F-BOX1/LOV KELCH PROTEIN2 family

In Arabidopsis, the ZEITLUPE/FLAVIN BINDING, KELCH REPEAT, F-BOX1/LOV KELCH PROTEIN2 (ZTL/FKF1/LKP2) family group of blue-light photoreceptors that deploy FMN as the chromophore is mainly involved in the regulation of the circadian clock and flowering (Somers *et al*., 2000; Baudry *et al*., 2010; Takase *et al*., 2011). Only one gene in tomato belonging to this family has been found, namely FKF1 (Fantini *et al*., 2019; Shibuya *et al*., 2021). Little is known about its biological function in tomato, but Shibuya *et al*. (2021) demonstrated high sequence identity between the *FKF1* genes in Arabidopsis and tomato, as well as a high conservation of the amino acids essential for its functioning, suggesting a similar role for FKF1 in the two species.

### UV-B RESISTANCE 8

Plants perceive UV-B light (280–315 nm), as well as short-wavelength UV-A light, by the UV-B Resistance 8 (UVR8) photoreceptor, which has no specific chromophore but absorbs UV-B light by its uniquely positioned tryptophan residues (Christie *et al*., 2012; Wu *et al*., 2012; Rai *et al*., 2021). The inactive UVR8 dimer protein is activated by UV-B light and dissociates into active monomers, a process that is reversed in the dark (Rizzini *et al*., 2011; Yang *et al*., 2021). Tomato harbours one UVR8 receptor (Tossi *et al*., 2019), which controls several processes including UV-B-induced seedling hypocotyl elongation (X. Liu *et al*., 2020), and it can complement the Arabidopsis *uvr8* null mutant (Dong *et al*., 2021). Similar to Arabidopsis, the tomato UVR8 receptor protects the plant against damaging UV-light by stimulating the production of UV-absorbing compounds (e.g. anthocyanins) (Li *et al*., 2018; X. Liu *et al*., 2020). Interestingly, UVR8 is also involved in accumulation of pigments in tomato fruit (e.g. chlorophyll, lycopene, and β-carotene), even in the absence of UV light (Li *et al*., 2018).

Tomato plants are very responsive to their light conditions, both in terms of spectrum and fluence rate. This is illustrated by their elaborate system of photoreceptors, characterized by distinct absorption spectra, and the specific developmental processes that they regulate. Since the discovery of the phytochromes in 1959, plant photoreceptor research has progressed significantly, and the diversity and functioning of many of these photoreceptors in tomato has been extensively studied. This has contributed to advances in commercial greenhouse production; however, our understanding of the roles of phototropins and the ZTL/FKF1/LKP2 family in tomato remains limited and warrants further research.

## Leaf and crop photosynthesis

### Light intensity and its fluctuations

Tomato leaves are exposed to fluctuating light conditions due to cloud movements, shading between leaves and from greenhouse infrastructure, and the switching of supplementary lighting on or off. light fluctuations reduce light-use efficiency, and optimizing photosynthesis to fluctuating conditions has been identified as a potential avenue to increase overall plant productivity (Kaiser *et al*., 2015; Acevedo-Siaca *et al*., 2020, 2021; Long *et al*., 2022; Shao *et al*., 2024). Ideally, photosynthetic organs should receive sufficient light energy to adequately drive photosynthesis and avoid light limitation, while simultaneously deploying sufficient non-photochemical quenching (NPQ) to protect the photosynthetic apparatus from photodamage (Lazzarin *et al*., 2024) without overly diverting electrons away from linear electron flow. Approaches that seek to optimize tomato photosynthesis to fluctuating light intensity need evaluation within the context of greenhouse production, in which light is sometimes diffused by the cover material (Holsteens *et al*., 2020), generating a unique light environment in which fluctuations are embedded within a background of more uniformly dispersed light. Fluctuating light can even be an issue within fully climate-controlled systems, such as vertical farming. For example, ventilation systems can cause movement and self-shading within the plant canopy (Gong *et al*., 2022; Agati *et al*., 2024), which can cause ‘micro-fluctuations’. To date, no work has focused on understanding these micro-fluctuations and whether they present a potential limitation to photosynthesis.

### Light spectrum and its fluctuations

The photosynthetic response to the light spectrum is traditionally defined in the region of photosynthetically active radiation (PAR; 400–700 nm) (McCree, 1971) but it has been shown to extend outside this range with less efficiency (Inada, 1976; Zhen *et al*., 2022; Sun *et al*., 2024). The CO_2_ assimilation rate under UV-A1 (350–400 nm) is lower than within the PAR region and varies between species and cultivars (Sun *et al*., 2024). Considering the small fraction of UV in both solar and artificial light, and its low efficiency in driving photosynthesis, it might not contribute significantly to CO_2_ assimilation. Furthermore, UV and blue light can induce photoinhibition, but again their proportions are often low in controlled environment agriculture (Nawrocki *et al*., 2021). Green light is absorbed less by leaves but penetrates deeper than red or blue light into the leaf and the canopy (Terashima *et al*., 2009; Liu and van Iersel, 2021); however, a meta-analysis has shown that it is similarly effective in promoting plant biomass as red and blue light together (Chen *et al*., 2024). Only under strong white light does extra green light absorbed by lower-canopy chloroplasts boost photosynthesis more effectively than extra red or blue light. Outside the PAR region, FR can stimulate photosynthesis due to the Emerson effect, especially under light with wavelengths that over-excite PSII (700–750 nm; Zhen and van Iersel, 2017; Zhen *et al*., 2022). Essential factors to consider when studying gas-exchange responses to light spectra are the interaction and the limitations between photosynthesis, stomatal conductance, and other processes that vary under different wavelengths (Vialet-Chabrand *et al*., 2021).

### Light direction

Tomato leaf anatomy varies greatly (Nakayama *et al*., 2023) and plays a key role in light propagation. The palisade mesophyll channels collimated light deeper into the leaf (Brodersen and Vogelmann, 2010), demonstrating that they are not simple light absorbers. The cellular structure influences light scattering, and incoming light with oblique angles reduces net photosynthesis partly due to increased reflection (Yates, 1981). Another reason is the weak penetration of low-angle light, which creates heterogeneous saturation of electron transport in the upper leaf layers and reduces photosynthesis rates (Brodersen and Vogelmann, 2010; Earles *et al*., 2017). The angle distribution of different light sources can therefore affect net photosynthesis rates, and it is not accounted for in most photosynthesis models (Berry *et al*., 2022). In addition to the light angle, illuminating the adaxial versus abaxial leaf side can alter both dynamic and steady-state photosynthesis responses (Wall *et al*., 2022), highlighting the asymmetric photosynthetic characteristics of leaves (Wang *et al*., 2023).

### Photoperiod

Extending lighting beyond 18 h causes interveinal chlorosis in tomato leaves and does not further increase yield (Velez-Ramirez *et al*., 2017a; Lanoue *et al*., 2019). The response of tomato plants to photoperiod length is genotype-dependent as at least one gene (*CHLOROPHYLL A/B BINDING PROTEIN 13*) has been identified that confers tolerance to injuries due to excessive light, such as during long or continuous photoperiods (Velez-Ramirez *et al*., 2014). The benefit increased carbon gain is limited by down-regulation in photosynthesis due to excess accumulation of carbohydrates and the early leaf senescence observed under extended photoperiods (Velez-Ramirez *et al*., 2017a). More work needs to be done in tomato plants to develop lighting strategies that maximize photosynthesis over the course of the photoperiod. One potential approach for applying continuous lighting is entraining the circadian clock of the plants by changing the light spectrum and intensity, and the temperature over a 24 h period, which limits carbohydrate accumulation and light injuries (Velez-Ramirez *et al*., 2017b; Lanoue *et al*., 2019; Marie *et al*., 2024). Using continuous lighting with low but varying intensity could be particularly useful to track daily energy price fluctuations while increasing yield.

### Light energy

In addition to driving photosynthesis, the input of light influences the energy balance and temperature of the leaf, thereby affecting various processes (Jones, 2013). The contribution of leaf temperature fluctuations to dynamic photosynthesis that results from light-intensity fluctuations is not well characterized. Most of the literature considers the temperature response of photosynthesis in steady-state conditions (Bernacchi *et al*., 2001), ignoring the potential dynamic effects. Photosynthesis consists of several temperature-dependent biochemical processes with different optima; this includes the induction of photosynthesis (Moore *et al*., 2021). An increase in temperature alongside increasing light intensity can enhance the photosynthetic rate during the initial stage of the induction process (Kang *et al*., 2020), and raises questions about what happens under continuously fluctuating light intensity. In addition, light-control regimes incorporating dynamic lighting applications can be used for decreasing electricity costs, shifting usage to periods with lower prices without negative consequences for the tomato plants (Ramírez *et al*, 2019).

Improving carbon fixation per photon is a key factor for crop production and can be achieved at different temporal and spatial scales. Light intensity and the spectrum during growth can be further fine-tuned to promote leaf anatomical changes that improve light capture and CO_2_ assimilation. Studies focusing on dynamically varying intensity, angle, and spectrum are still lacking in the literature but represent an untapped opportunity for improving tomato yield.

## Plant morphology and light interception

Greenhouse tomato plants are commonly grown in rows, which not only facilitates crop management and harvest, but also allows deeper light penetration inside the canopy. Light intensity decreases exponentially from the top to the bottom of the canopy, following the Beer–Lambert Law and characterized by the light-extinction coefficient (Monsi and Saeki, 2005; Heuvelink *et al*., 2020), which is closely dependent on the plant architecture and solar elevation as well as light direction. Simulations have shown that an ideotype architecture of greenhouse-grown tomato with long internodes and long and narrow leaves can substantially increase crop photosynthesis due to improved total light interception (Sarlikioti *et al*., 2011).

### Light distribution and interception

Light extinction in a canopy is spectrum-dependent. For example, FR and green light penetrate deeper at both leaf and canopy level compared to red and blue light (Smith *et al*., 2017; Kaiser *et al*., 2019b; Stamford *et al*., 2023). For tomatoes grown in a greenhouse where 71% of the light is made diffuse, annual crop photosynthesis is improved by 7.2% (Li *et al*., 2014); however, Holsteens *et al*. (2020) found the positive effects of diffuse light only to be significant at high light intensities. The positive effects of diffuse light are due to more uniform horizontal light distribution, more uniform vertical light distribution, higher leaf photosynthetic capacity (probably due to acclimation of lower leaves to higher light intensity), and extra leaf area expansion (Li *et al*., 2014). Furthermore, placing LED lights in within the canopy improves light distribution and the photosynthetic capacity of leaves lower in the canopy (Gómez and Mitchell, 2016), and consequently increases tomato yield by up to 17% compared to LED placement solely above the canopy (Schipper *et al*., 2023; Stamford *et al*., 2023; Schouten *et al*., 2024).

### Morphology

Tomato plants grown in shade (characterized by low light intensity and low red:FR) produce long internodes and petioles and have reduced shoot branching, which are typical characteristics of the shade-avoidance syndrome (SAS) (Chitwood *et al*., 2015; Ji *et al*., 2019). Phytochromes are the dominant photoreceptors to regulate SAS in tomato, but cryptochromes can also play a role (Schrager-Lavelle *et al*., 2016; Liu *et al*., 2018; Fantini *et al*., 2019). Many important downstream light-signalling components have also been reported in tomato, for example, via PHYTOCHROME-INTERACTING FACTOR transcription factors and growth-related hormones such as auxin, gibberellic acid, brassinosteroids, and abscisic acid (Cagnola *et al*., 2012; Liu *et al*., 2018, 2021; Xie *et al*., 2022; Gautrat *et al*., 2024). Heating effects of lighting on plant architecture are strikingly similar to SAS (Gautrat *et al*., 2024). Our knowledge on spatial perception and regulation of light signals in the tomato plant is rather fragmented. Interestingly FR perceived by adult plant parts can also influence the morphology of young developing tissue (van der Meer *et al*., 2023). Furthermore, Ernesto Bianchetti *et al*. (2018) provided evidence for the regulation of processes in the tomato fruit by phytochromes localized within them. This was supported by Okello *et al*. (2015) who showed that illumination of fruit stimulates cell division, and by Guan and Janes (1991) who concluded that the sink strength of fruits grown *in vitro* increases when they are illuminated. Gautrat *et al*. (2024) have also stressed the need for research on the regulation of light cues spatially within the plant.

**Fig. 4.**
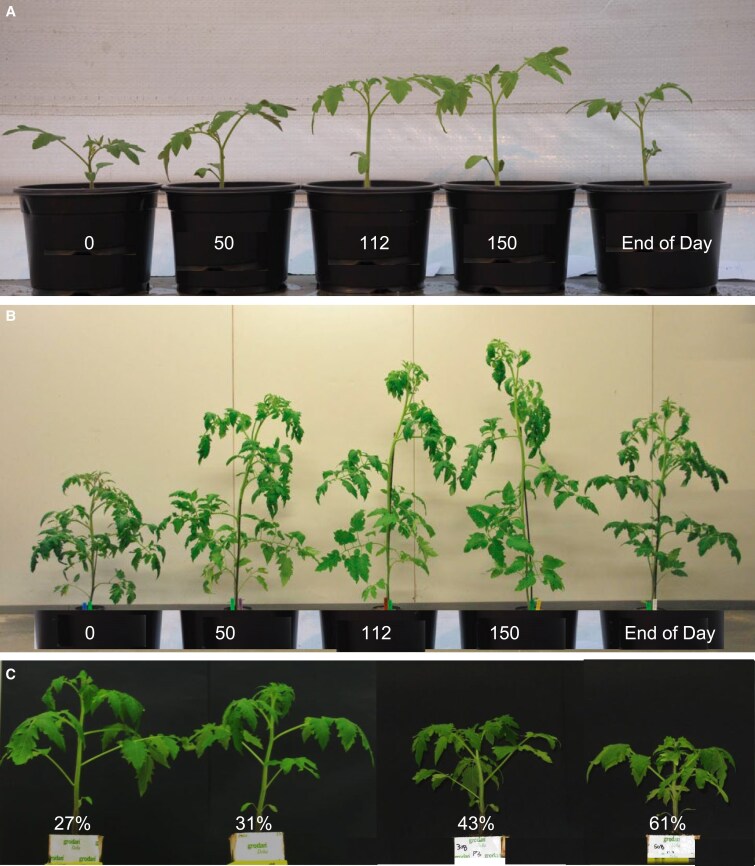
Effects of different levels of additional far-red (FR) radiation or different fractions of blue light on the growth of tomato plants. (A, B) At 15 d after sowing, plants were treated with 0, 50, 112, or 150 μmol m^−2^ s^−1^ FR (resulting in phytochrome photostationary state values of 0.88, 0.80, 0.74, 0.70, respectively). FR was added to a background of 150 μmol m^−2^ s^−1^ red plus blue light. In addition, 17 μmol m^−2^ s^−1^ FR was added for 15 min as an ‘end-of-day’ (EOD) treatment. Plants were photographed after (A) 7 d or (B) 28 d. From Kalaitzoglou *et al*. (2019). (C) Plants were grown for 23 d under solar-like white light from plasma lamps containing 27% blue, and 0, 5, 30, or 50% of the white light was replaced by blue, resulting in blue-light fractions of 27, 31, 43, and 61%, respectively. The total photosynthetically active radiation was 100 μmol m^−2^ s^−1^, and the light treatments started at 7 d after sowing. From Kalaitzoglou *et al*. (2021).

Photoreceptor signalling pathways that induce or mitigate SAS offer the possibility of using different light spectra to regulate plant architecture through LEDs. For example, supplementary FR induces internode elongation (Fig. 4A, B) and alters leaf positioning and plant architecture, which could improve light distribution within the canopy (Kalaitzoglou *et al*., 2019; Zhang *et al*., 2019; Ji *et al*., 2020). When tomato plants perceive an increasing fraction of blue, they often have shorter internodes, and smaller and thicker leaves (Fig. 4C), which is opposite of SAS (Dieleman *et al*., 2019; Kalaitzoglou *et al*., 2021; Stamford *et al*., 2023). This is probably mostly due to the increase of blue light intensity being sensed by cryptochromes, but a change in the phytochrome photostationary state might also play a role (Sager *et al*., 1988; Kong and Zheng, 2024). Kaiser *et al*. (2019a) varied the percentage of blue supplemental light (in a red background) as 0, 6, 12, and 24% while keeping total photosynthetically active radiation constant and found that total biomass and fruit yield increased with the addition of blue light to an optimum between 6–12%. Decreased biomass at low (0%) blue light was probably caused by decreased photosynthetic light-use efficiency. Conversely, decreased biomass at high (24%) blue light was probably caused by reductions in canopy light interception. Kusuma *et al*. (2023) applied supplemental LED lighting to greenhouse tomatoes and found that when the red percentage of white LEDs increased from 38% to 95% (at the same time percentage blue decreased from 19% to 5% and percentage green decreased from 43% to 0%) the fruit fresh yield decreased by 13% and total plant dry mass by 7.1% in one cultivar, whereas there were no significant effects in another cultivar. With an increasing fraction of green light, tomato plants tend to have taller stems and thinner leaves (Dieleman *et al*., 2019; Kaiser *et al*., 2019b), which could be modulated by both cryptochromes and phytochromes (Folta and Maruhnich, 2007; Battle *et al*., 2020; Zhang *et al*., 2021, 2022). Adding UV-A1 to red light is reported to induce steeper leaf angles, flatter leaves, and longer stems along with increasing biomass in young tomato plants compared with adding blue light (Y. Zhang *et al*., 2020). This is probably dependent on UV-A-mediated auxin transport and cell-wall acidification (Zhang *et al*., 2024), but how photoreceptors are involved is still unknown.

Light also plays an important role in the energy balance of plant organs. A higher photosynthetic photon flux density (PPFD; 400–700 nm, μmol m^−2^ s^−1^) results in an increase in absorbed irradiance, and hence a higher energy input. This leads to a temperature rise. For instance, Savvides *et al*. (2013) observed that temperature of meristems of tomato was lower than the air temperature in darkness, whereas under 850 µmol m^−2^ s^−1^ PAR provided by high-pressure sodium lamps the meristem temperature was 2–4 °C higher than air temperature, depending on the wind speed. Such a difference in the temperature as a result of turning lamps on can have large consequences for plant growth and development (Savvides *et al*., 2017).

In summary, the light environment not only affects light interception directly but also indirectly through modifying plant architecture. Tomato architecture is regulated by multiple light cues, but how the integration of information occurs is largely an open question (Gautrat *et al*., 2024). Functional–structural plant models of tomato that combine 3D plant architecture and ray tracing might help in understanding the relationships between plant architecture and light interception and distribution, and the consequences for crop photosynthesis (Shin *et al*., 2021; van der Meer *et al*., 2021; Schipper *et al*., 2023). Further research is still required for a systematic understanding of the relationship between canopy structure and tomato growth, by incorporating spatial and temporal variations in plant physiological responses such as dynamics in photosynthesis, and sugar metabolism as well as sink–source relationships.

## Carbon assimilate partitioning and fruit growth

Carbon assimilate partitioning is strongly influenced by light quantity, quality, and photoperiod, and it occurs both at the organ level (dry mass partitioning among organs) and the biochemical level (partitioning among carbon pools). Source-to-sink carbon partitioning in tomato has been reviewed by Osorio *et al*. (2014).

### Partitioning between organs

**Fig. 5.**
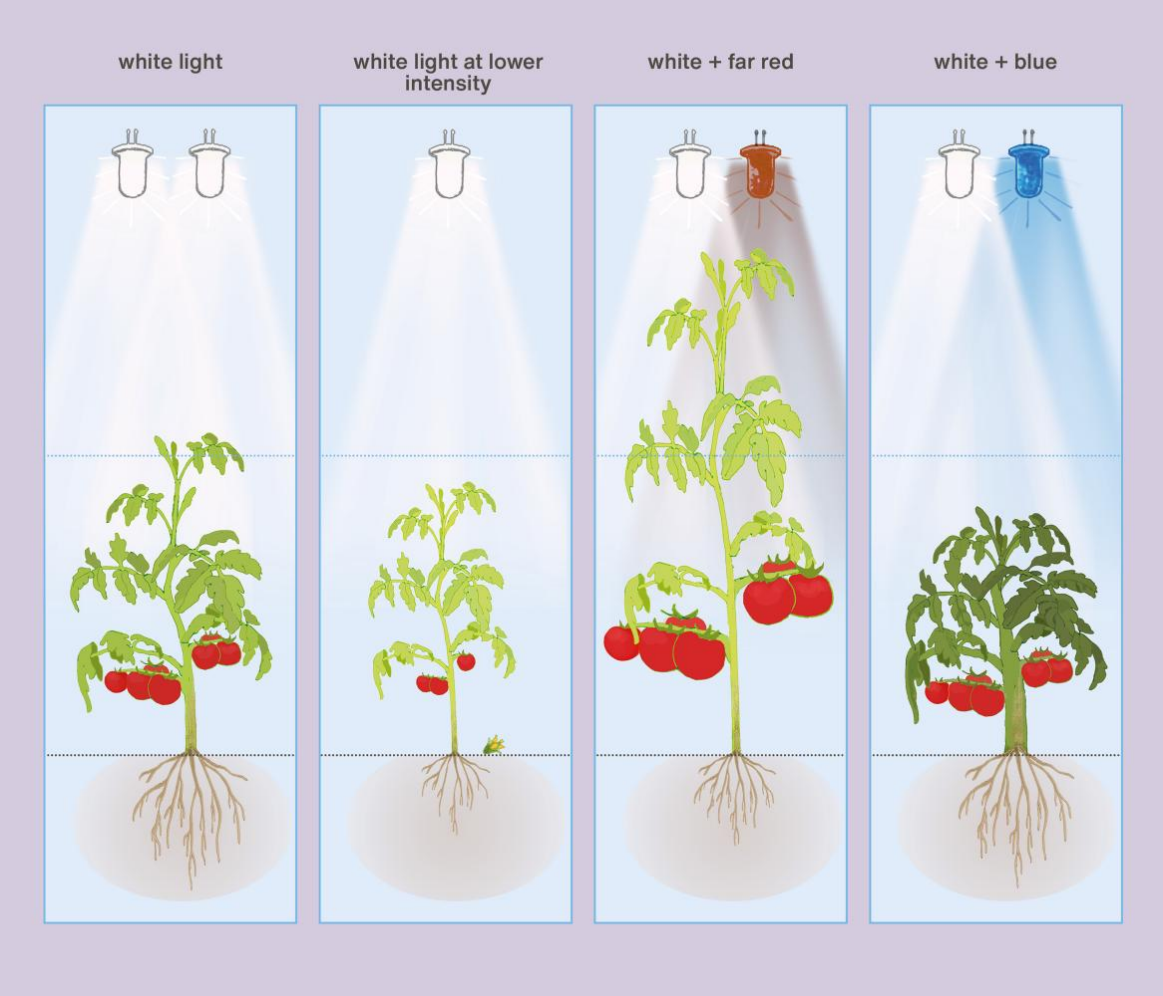
Schematic illustration of the responses of carbon assimilate partitioning among plant organs to different light regimes in fruiting tomato plants. Low light intensity can lead to fruit abortion, resulting in a low fraction of carbon assimilates partitioned into fruits. Low light also decreases the fraction of assimilates partitioned to the roots. Far-red (FR) increases the fruit sink strength, resulting in an increase in the assimilate partitioning to the fruits. FR also increases the relative amount of assimilate partitioned to stems at the expense of leaves and roots. Blue light results in more compact plants, while strong effects on carbon partitioning have not yet been documented.

To quantitatively explain dry mass (DM) partitioning at the organ level, it is crucial to understand the concept of sink strength, which is the intrinsic capacity of each organ to attract photosynthetic assimilates (Marcelis, 1996; Box 1). Taking the fruit as example, the fraction of DM partitioned is proportional to the ratio between the total sink strength of all the fruits relative to the total sink strength of the whole plant (Heuvelink, 1996). Light may influence DM partitioning to fruits by affecting the sink strength of an individual fruit or by affecting the total fruit number (Fig. 5). For example, Ji *et al*. (2019) reported that FR during growth increased the fraction of DM partitioned to fruits. This was caused by an increased sink strength of individual fruits due to enhanced sugar metabolism and transport, as was demonstrated by the expression of corresponding genes (Ji *et al*., 2020) While the appearance of the first flower can be affected by an interruption of the night by a short light period (Cao *et al*., 2016) and by light level (Savvides *et al*., 2014), the appearance rate of fruit trusses after the first flower is hardly influenced by the latter (Heuvelink and Marcelis, 1996). A low light level can also decrease DM partitioning to fruits as a result of a reduction in fruit number due to flower and fruit abortion (Kinet, 1977; R. Li *et al*., 2021).

Box 1. **Fruiting plants are source-limited, young plants can be sink-limited**

The balance between source and sink strength is important for growth, dry matter partitioning, and fruit quality in tomato plants (Bertin *et al*., 2002; Osorio *et al*., 2014). Sink strength of an organ can be defined as its ability to attract assimilates; sink strength of a whole plant is the sink strength of all organs of the plant together (Marcelis, 1996). In tomato plants, fruits are by far the most important sink organs on the plant. Source strength can be defined as the photoassimilate production of the plant. When source strength is larger than sink strength, the growth of the plant is sink-limited; when sink strength is larger than source strength the plant is source-limited. When sink-limited, feedback inhibition of photosynthesis can occur (Paul and Foyer, 2001) and plant growth cannot be increased by more light. When source-limited, tomato fruits will be small and often of poor quality (Karpe *et al*., 2024).

In the fruiting stage, increasing the planting density leads to an increase in sink strength of the whole crop (as more stems per m^2^ leads to more fruit trusses per m^2^). However, the planting density in the fruiting stage hardly affects source strength as almost all the light is intercepted except when a very low planting density is used (Heuvelink, 1995). Consequently, in commercial high-tech greenhouses where plants are grown at high density, they are almost always source-limited (Li *et al*., 2014). Fruiting plants are only likely to be sink-limited when they are grown separately with no competition for light by neighbouring plants.

In contrast to fully fruiting tomato plants, young vegetative plants (or those at the very beginning of the fruiting stage) that do not have many sink organs can be sink-limited, depending mainly on daily light integral, planting density, temperature, and CO_2_ concentration (Li *et al*., 2014). Feedback inhibition of photosynthesis can occur when plants are sink-limited (Paul and Foyer, 2001). Hence, studies of photosynthesis on young vegetative plants might have limited predictive value for fruiting plants if no attention is paid to sink limitation.

Light signals also regulate the DM partitioning between above- and below-ground organs. The concept of functional equilibrium (Brouwer, 1983) has often been used to explain that the growth of shoot organs is relatively favoured over roots when light is reduced. Furthermore, FR promotes DM partitioning towards shoots over roots in tomato (Ji *et al*., 2021).

### Partitioning between structural and non-structural carbon

Carbon assimilates can be actively allocated to structural growth (e.g. cellulose, hemicellulose, lignin) or storage compounds, referred to as non-structural carbohydrates (NSCs), such as sugars, starch, oligosaccharides, polysaccharides, lipids, and amino acids (Hoch *et al*., 2003). This allocation is an active process, allowing plants to prioritize storage at the expense of structural growth (Dietze *et al*., 2014). Unlike a passive process driven solely by the balance between carbon supply from photosynthesis and demand for growth and respiration, active assimilate partitioning reflects the dynamic response of the plant to environmental conditions (Kozlowski, 1992). This differentiation has critical implications as prioritizing allocation towards storage comes at the expense of structural growth, therefore potentially reducing leaf area and limiting light capture, which is critical in particular during the exponential growth phase of tomato plants. The two main mechanisms actively regulating carbon storage in plants are the partitioning of assimilates between soluble sugars and starch and the remobilization of this storage compound (Zepeda *et al*., 2023).

Manipulating the light environment influences carbon assimilate partitioning between soluble sugars and starch, with starch partitioning being strongly dependent on photoperiod and weakly on light quantity. Long days result in less carbon allocated to starch, while shorter days promote increased storage (Pilkington *et al*., 2015; Mengin *et al*., 2017). This inverse relationship between starch accumulation and the length of the photosynthetic period has been observed across various species (Chatterton and Silvius, 1980; Grange, 1985; Graf *et al*., 2010) including tomato (Logendra and Janes, 1992), even when daily light integrals remain constant.

Shorter photoperiods at a constant daily light integral result in thinner leaves despite maintaining similar leaf areas, suggesting reduced allocation to structural growth (Chatterton and Silvius, 1980). Vegetative tomato plants exposed to varying light intensities (200–400 µmol m^−2^ s^−1^) and temperatures (18–28 °C) over 20 days while keeping the light and temperature integrals constant show similar DM accumulation (Zepeda *et al*., 2022). However, under sink-limited conditions (high light intensity relative to temperature), the plants allocate a higher fraction of biomass to NSCs at the expense of structural growth, compared with source-limited conditions (low light intensity relative to temperature).

This recent understanding of how light regulates carbon assimilate partitioning between different organs and different forms of carbon provides exciting possibilities to actively steer the partitioning of carbon within the plant. Light can increase the fraction of biomass partitioned to fruits via increasing the sink strength of the individual fruits (e.g. additional FR increases the sink strength) as well as by increasing the total number of fruits (light quantity stimulates fruit set and therefore the number). The root:shoot ratio also strongly responds to light, with smaller ratios at low light levels or with additional FR. Such light control can potentially be applied in a smart and dynamic way to not only shape the phenotype of the tomato plant but also to maximize the yield and quality of the fruits.

## The interplay of light, water, and nutrients

### Plant–water relations

Water movement in plants operates along the soil–plant–atmosphere continuum (SPAC), driven by transpiration. This process ensures plant hydration, mediates nutrient transport, and regulates plant temperature. Light plays a critical role in modulating transpiration rates, stomatal conductance (*g*_s_), and hydraulic conductance, which together determine water transport efficiency in plants.

Blue light plays a dominant role by stimulating phototropins, leading to stomatal opening and increased transpiration rates (Van Ieperen *et al*., 2012; Barillot *et al*., 2021). This effect can, in turn, reduce leaf elongation rates, as has been speculated for tall fescue (Barillot *et al*., 2021). In contrast, FR decreases *g*_s_ in tomato, resulting in lower transpiration rates and conserving water per unit leaf area (Kalaitzoglou *et al*., 2019; Wassenaar *et al*., 2022); however, higher FR levels often increase leaf area (see below), which can increase whole-plant transpiration. Chen *et al*. (2024) conducted a meta-analysis that showed that green light increases intrinsic water-use efficiency (the ratio of net photosynthesis to stomatal conductance) by reducing *g*_s_ without significantly affecting photosynthesis. These light-driven adjustments in *g*_s_ highlight the importance of spectral composition in managing water use in controlled environments.

Leaf hydraulic conductance (*K*_leaf_) serves as a key determinant of water transport efficiency across the SPAC, with its response to light intensity and spectrum playing a central role (Grunwald *et al*., 2024). While studies have demonstrated a positive correlation between light intensity and *K_l_*_eaf_ in many tree species, with sun-leaves exposed to higher irradiance showing several-fold increases in *K_l_*_eaf_ and greater plasticity to fluctuating light conditions compared with shade-leaves (Õunapuu-Pikas and Sellin, 2020), research on herbaceous plants remains limited, including tomato. In other herbaceous crops distinct spectral effects on *K*_leaf_ have been found, in particular for the ratio of blue:red light, highlighting the need for further research on tomato. In several species, the absence of blue light leads to a 60–70% reduction in *K*_leaf_ (Grunwald *et al*., 2024). In cucumber, monochromatic blue light induces the highest *K*_leaf_, followed by a combination of blue and red light (30:70), while monochromatic red light results in low *K*_leaf_ (Savvides *et al*., 2012; Van Ieperen *et al*., 2012). Savvides *et al*. (2012) also demonstrated coordination between *K_l_*_eaf_ and *g*_s_ across light spectra, with leaves grown without blue light showing not only lower *K*_leaf_ but also lower *g*_s_, leading to reduced photosynthesis. In addition to this long-term acclimation of *K*_leaf_, rapid responses of *K*_leaf_ to light have also been reported. These studies highlight changes in water permeability of out-of-xylem tissues, mediated by aquaporin activity in response to blue signals in vascular bundle-sheath cells and mesophyll cells (Shatil-Cohen *et al*., 2011; Sade *et al*., 2014; Grunwald *et al*., 2024).

### Calcium uptake and distribution

A close correlation between irradiance and transpiration is often observed (Van Ieperen and Madery, 1994). Uptake of immobile calcium (Ca^2+^) is closely linked to water relations, as transpiration-driven flow facilitates its transport. However, in tomato, high light intensities can induce high transport of water (and therefore Ca^2+^) to the leaves instead of fruits, while simultaneously increasing fruit growth; this creates a Ca^2+^ imbalance in the fruits resulting in the physiological disorder blossom-end rot (BER) (Ho *et al*., 1993; Taylor and Locascio, 2004). Lower light intensities can rebalance water distribution within the plant, reducing the incidence of BER (Ilić *et al*., 2012); however, low PPFD and high humidity during leaf development can lead to calcium deficiency in the leaves (Airman and Houter, 1990).

### Fruit water balance

Tomato fruit development is highly sensitive to light intensity, with high intensities reducing xylem water influx into fruits while increasing phloem contributions (Hanssens *et al*., 2015). The implications of this are that high light intensity can increase fruit quality by increasing the flow of sugar into the fruits (see below); however, tomato fruit dry matter content has been shown to be unaffected by PPFD (Kläring and Krumbein, 2013).

### Cell wall structure and turgor-driven growth

In addition to its role in water transport, light plays a crucial role in modulating cell wall structure and extensibility, both of which are important for cell expansion. When positive turgor pressure in living cells surpasses a critical wall-yielding threshold, it drives the extension of the cell wall, facilitating growth, a phenomenon known as turgor-driven growth (Steppe *et al*., 2006, 2015; De Swaef and Steppe, 2010). Monochromatic blue and white light induce thicker, more rigid cell walls in tomato seedlings, largely due to increased calcium and pectin levels (Falcioni *et al*., 2020). In contrast, FR and darkness result in thinner cell walls, while monochromatic red or green light produces intermediate thickness (Falcioni *et al*., 2020). These structural changes towards a more rigid cell wall limit cell expansion and turgor-driven growth but enhance mechanical strength, supporting the plant under high-light conditions. The thinner, more flexible cell walls produced under FR and in darkness facilitate cell expansion and turgor-driven growth. These findings highlight the complex interplay between light, cell wall properties, and turgor-driven growth.

Light plays a pivotal role in regulating water transport and nutrient dynamics by influencing transpiration, stomatal conductance, hydraulic conductance, and cell wall properties. However, significant knowledge gaps remain regarding its immediate and long-term effects on *K_l_*_eaf_. Future research should prioritize elucidating the fundamental principles driving water use efficiency, turgor-driven growth, and the development and quality of tomato fruits.

## Fruit quality and ripening

Tomato is a climacteric fruit that requires ethylene to ripen (Y. Liu *et al*., 2020). During ripening, the fruit softens, starch is converted into simple sugars, and acids and aroma compounds are synthesized, all of which contribute to the characteristic flavour of tomatoes (Powell *et al*., 2012; Lu and Zhu, 2022). Moreover, the fruit transitions from green to red due to chlorophyll degradation and lycopene biosynthesis (Schouten *et al*., 2014). There is a post-harvest decline in tomato quality; however, specific pre-harvest lighting conditions can improve quality (see Table 1 for an overview of the literature).

**Table 1.** Effects of light treatments on tomato quality

Experimental conditions					Quality parameters	References
Additional light*	Daily light intregral (mol m^−2^ d^−1^)	PPFD (µmol m^−2^ s^−1^)	Photoperiod (h/d)	Control/Background		
Inter-canopy R+B at daytime or nighttime	7	165 (mid-canopy)	12 daytime (04.00–16.00 h) or 12 nighttime (22:00–10:00)	Sunlight	*In summer*: ↑ TSS in daytime fruit compared with control and nighttime *In winter*: ↑ TSS and ↑ Ascorbic acid in daytime and nighttime compared with control fruit	Tewolde *et al*., 2016
Inter-canopy R (660 nm) + B (450 nm), ratio 20:80	27	420	18 (04.00–22.00 h)	HPS 420 µmol m^−2^ s^−1^	TSS; TA; Minerals (↑ Ammonium, ↑ Sodium)	Paponov *et al*., 2020
FR: 0, 30, and 50 µmol m^−2^ s^−1^	9 (control), 10, 12	150 (control), 180, 200	16	Sunlight+red and blue light	*Ripe fruit*: Colour. ↑ Firmness at harvest, ↓ Decay of fruit after 0, 10, and 15 d at 4 °C, and 20 d at 20 °C	Affandi *et al*., 2020
R (657 nm), B (457 nm), and R+B, ratio 3:1	13	300	12	White	Colour change: RB>R>W and B; Firmness: RB<W and R<B; Lycopene and sugars: RB>R>W>B	Y. Li *et al*., 2021
Top and intra-canopy B: 0, 12, and 24% (445 nm)	12	123 (top)+86 (intra-canopy)	16	Sunlight+red light (665 nm)	↓ Red colour, ↑ Firmness, and ↓ Decay of red fruit grown under 12% blue light and stored for 20 d at 4 °C; Weight loss; Ascorbic acid	Affandi *et al*., 2022
RB: R (660 nm) + B (465 nm), ratio 3:1; RB + 40 µmol m^−2^ s^−1^ Far-red (730 nm) for the whole photoperiod (FR) or for 30 min at end of day (EOD)	10 (RB) and 12 (FR and EOD)	170 (RB), 210 (FR and EOD)	16 (08.00–12.00 h)	Sunlight	*Immature fruit*: ↑ Hardness in RB compared with control, ↑ TSS in RB compared with control and EOD, ↓ β-carotene in EOD; *Ripe fruit*: ↑ Total phenolics in RB, FR, and EOD compared with control, ↓ Weight loss of FR and EOD, ↑ β-carotene in RB compared to EOD, ↓ Severe decay in RB; Lycopene; Total antioxidant capacity; Acidity	Appolloni *et al*., 2022
R, G, and B with HPS lamps (ratio 4:20:1) or LED lamps, ratio 5:4:1	5 (HPS) and 7 (LED)	143 (HPS) and 176 (LED)	10.5	Sunlight	Colour change: HPS>LED>control; Ripe fruit: ↑ TSS and TA in LED compared with control	Yang *et al*., 2022
R and B (ratio 7:2) in the morning or in the evening	14 (control) and 16 (R+B) Morning: 3 h R+B (05.00–08.00 h) then 10 h white light Evening: 10 h white light (08.00–18.00 h) then 3 h R+B	Control: 400 (10 h) Morning: 51 (3 h) followed by 400 (10 h) Evening: 400 (10 h) followed by 51 (3 h)	Control: 10 Morning and evening: 13	White light 10 h/Dark	*Morning*: ↑Ascorbic, oxalic, tartaric, total organic acids, and total phenols; *Evening*: ↑ Fructose, glucose, sucrose, total sugars, and flavonoids compared with control and morning treatment; Carotenoids and amino acids: Morning>Evening>Control; ↑ Calcium; Changed volatile profile	Wang *et al*., 2022
Red (660 nm) and white, ratio 1:1; red and white, 3:2; red, white, and blue (460 nm), 1:1:0.5, 10 d before harvest	9	200	12	White light/Dark	Colour change: RWB>R3W2>Control>R1W1; Firmness; Lycopene; Carotenoids	Yan *et al*., 2023
R, G, and B at low or high intensity	7 or 13	150±10 or 300±10	12	White light (R:G:B=19:48:33)	Soluble sugars: ↑ Red high and Blue low intensity; TA: blue and red>green and white; Ascorbic acid: ↑ Blue and red compared with control and green; B-carotene: ↑ Red high intensity; Lycopene: ↑ Blue and green high intensity; Changed volatile profile	Fan *et al*., 2023
Top, intracanopy, or top+intra-canopy. R (660 mm), B (460 nm), and FR (730 nm), ratio 1:0.29:0.14	6	120	14 (06.00–20.00 h)	Sunlight	Ascorbic acid: Top+intra-canopy>Intra-canopy>Top>Control; Lycopene: Intra-canopy>Top+Intra-canopy and Top>Control	Ziaei *et al*., 2024
R (667 nm), W, and R+B (450 nm), ratio 3:1 or 9:1	12 (early stage) and 17 (later stages)	200 (early stage) and 300 (later stages)	16	White	TSS: White>R3B1; Acidity	Ke *et al*., 2024
FR during the whole, first half, or second half of photoperiod	3, 2, and 2	226±43 (control); 226±1 (FR whole); 227±3 (FR first half); 226±1 (FR second half)	16, 8	Red and white (R:G:B=88:5.5:5.5)	↑ TSS in FR first half compared with control; ↑ pH in FR whole and FR second half	Vincenzi *et al*., 2024

^*^Lighting is supplied at the top of the canopy unless otherwise specified.

B, blue; FR, far-red; R, red; W, white; HPS, high-pressure sodium; LED, light-emitting diode; TA, titratable acidity; TSS, total soluble solids. ↑, significant increase relative to control; ↓, significant decrease relative to control. Comparative rankings among treatments are indicated by > and <.

### Regulation of ripening

The use of monochromatic lights in addition to natural or white light has facilitated the elucidation of how light promotes ripening (Xie *et al*., 2019; J. Zhang *et al*., 2020). Additional red and blue light increases the red colouration of tomato by increasing expression of genes for phytochromes (*PHYA*, *PHYB1*, *PHYB2*, *PHYF*) and cryptochromes (*CRY1a*, *CRY3*), thereby initiating downstream signalling pathways (Xie *et al*., 2019; J. Zhang *et al*., 2020; Wang *et al*., 2021b; Yan *et al*., 2023). Additional red and blue light stimulates *PSY1* and *ZDS*, which encode a phytoene synthase and a ζ-carotene desaturase, respectively. These are crucial for lycopene biosynthesis, and down-regulate *CYCB*, a lycopene β-cyclase involved in lycopene degradation (Xie *et al*., 2019; Yan *et al*., 2023). Supplementing sunlight with red light at the mature green and breaker stages down-regulates genes encoding ethylene receptors (*ETR3*, *ETR4*, *ETR6*) and up-regulates biosynthesis genes (*ACS2*, *ACS4*, *ACO1*), leading to enhanced ethylene signalling and accelerated ripening (J. Zhang *et al*., 2020). Additionally, red light induces ripening-related genes such as *RIN*, *NOR*, and *CNR*, which contribute to ethylene production and carotenoid accumulation (Fantini *et al*., 2019; J. Zhang *et al*., 2020; Yan *et al*., 2023). Blue light complements this effect by stimulating ethylene release (He *et al*., 2022). Mixed red and blue light synergistically increases melatonin and ethylene levels, promoting lycopene accumulation (Y. Li *et al*., 2021). Furthermore, potassium transporters and ABA signalling are up-regulated by supplementary red and red plus blue light, leading to faster red colouring (Wang *et al*., 2021a).

### Chilling injury tolerance

An interesting light effect is its influence on improving resistance to chilling injury (CI), a common problem in cold storage that is characterized by discoloration and early decay (Farneti *et al*., 2012). Affandi *et al*. (2020, 2022) explored the effects of adding FR or changing the blue fraction in red–blue LED lighting (0, 12, 24%) during cultivation in a greenhouse and subsequent storage of the fruits at a CI-inducing temperature (4 °C). Additional FR and the intermediate fraction of blue light (12%) lowered decay by reducing weight loss and softening and red colour during cold storage, respectively, compared with treatments without FR or with 0% and 24% blue lightAdditional FR lowered decay by reducing weight loss and softening in fruits compared with those grown without FR, while the intermediate fraction of blue light (12%) resulted in fruits that exhibited a faster loss of red colour during cold storage compared with fruits grown with 0% and 24% blue light. However, Appolloni *et al*. (2022) did not observe significant effects of FR on CI.

### Metabolites

The contents of metabolites such as carotenoids and ascorbate can be increased by supplemental light (Tewolde *et al*., 2016; Appolloni *et al*., 2022; Wang *et al*., 2022; Ziaei *et al*., 2024). Increased light intensity enhances photosynthesis, promoting the accumulation of soluble carbohydrates and positively modulating the D-mannose/L-galactose and phenylpropanoid pathways, thereby increasing ascorbate and phenylpropanoid biosynthesis (Gautier *et al*., 2008, 2009; Ntagkas *et al*., 2018, 2019). Light-responsive genes encoding key enzymes in the ascorbate biosynthesis pathway (*GPP* and *VTC2*) are down-regulated by shading in red tomatoes but acorbate levels are not significantly affected (Massot *et al*., 2012).

### Flavour

The effects of light spectrum, intensity, and timing on total soluble solids (TSSs), including sugars and organic acids, remains a subject of debate. However, it is generally observed that TSSs increase under supplemental light, although the magnitude and consistency of this effect varies across studies (Tewolde *et al*., 2016; Fanwoua *et al*., 2019; Paponov *et al*., 2020; Appolloni *et al*., 2022; Yang *et al*., 2022; Ke *et al*., 2024; Vincenzi *et al*., 2024). Fanwoua *et al*. (2019) proposed that the positive effect of additional FR on glucose and fructose levels in tomatoes grown in greenhouses results from higher sugar production and reduced pericarp water content. Aroma volatiles can be changed in tomatoes grown with supplemental red and blue in the evening and in the morning (Wang *et al*., 2022).

### Texture

There is limited research on the impact of light on texture. Fruits from plants grown solely under blue light softened less rapidly during ripening compared to those grown under white, red, and red plus blue (Y. Li *et al*., 2021). Tomatoes grown under supplemental LED light consisting of 12% blue (88% red) were firmer than in the 0% blue (100% red) treatment (Affandi *et al*., 2022). Conversely, cultivation with supplemental combined red, blue, and white light results in softer fruit than those cultivated under white or white and red lighting (Yan *et al*., 2023). A negative correlation between fruit firmness and leaf mesophyll Ca^2+^ in plants irradiated with high light intensity of a broad spectrum has been proposed (Babla *et al*., 2023).

Increasing the light intensity during cultivation improves both the nutritional value and the flavour of fruits, through increasing the contents of ascorbate, carotenoids, sugars, and volatiles. Elucidating the light regulation of metabolic pathways enables the identification of targets for breeding and lighting strategies. Further research into spectral effects on quality parameters and light-mediated mechanisms of the regulation of texture would help tailor lighting strategies for improved fruit quality.

## Conclusions and outlook

Many studies have been conducted on the influence of photoperiod, intensity, spectrum, directionality, and energy of light. These aspects all play a part in regulating tomato plant morphogenesis, light interception, photosynthesis, source–sink interactions, assimilate partitioning, fruit set, fruit development, and plant–water relations, which collectively control plant growth and fruit quality. Combined functioning and cross-signalling of several photoreceptors regulate growth, development, and acclimation to environmental changes. Although the effects of light spectrum have been frequently studied, regulation of physiological processes by the spectrum is still missing in simulation models for crop growth and development. Storage of non-structural carbohydrate is most often considered a passive process; however, it is an active process that reflects the dynamic response of the plant to environmental conditions, with critical implications for growth. Most photobiology studies are based on young vegetative plants but further research is needed on fruiting tomato plants, which are predominantly source-limited whereas young plants are mostly sink-limited, potentially resulting in different responses. Most experimental work adheres to the ‘*ceteris paribus*’ principle: vary one factor and keep all others constant. This approach is very valuable, but in addition more studies are needed on the interactions between light spectrum, light intensity, temperature, and possibly other environmental and crop management conditions (Box 2). Fluctuating rather than constant conditions should receive more attention and will provide knowledge of the extent and time-frame of acclimation processes. Such research can further deepen our understanding of the physiological regulation of processes in the plant. Current knowledge on the spatial perception of light within plants and whether the responses are local or systemic remains incomplete and deserves more attention. Future research should prioritize exploring the interactions between light intensity and spectral composition to uncover the fundamental principles driving water use efficiency, turgor-driven growth, and the development and quality of tomato fruits. Elucidating the light regulation of metabolic pathways would enable the identification of targets for breeding and help tailor lighting strategies for improved fruit quality. The integrated study of light regulation of different physiological processes with their feedback and feedforward control mechanisms at the whole-plant level will further advance our understanding of tomato plant growth, development, and fruit quality.

Box 2. **Challenges in designing photobiology experiments**

*Monochromatic light*

In many studies the role of an individual colour (narrow waveband) is examined by growing plants under this colour alone, for example solely red light compared with growing under a different spectrum or in darkness. Such studies can have little relevance for understanding the physiology of plants under mixtures of colours. For example, increasing the fraction of blue light typically leads to compact plants (Fig. 4), but monochromatic blue light often results in elongated plants (Kim *et al*., 2014; Kong and Zheng, 2024). When added to a broad spectrum, red light can be the most efficient colour for photosynthesis, but growing plants solely under red can lead to the ‘red light syndrome’ in which photosynthesis is severely impaired, as shown for cucumber (Trouwborst *et al*., 2016). Furthermore, whilst observations made under monochromatic red light can be the result of the red light, they can also be the consequence of the absence of other colours, such as blue.

*The impossibility of studying the specific effects of only one colour*

Varying the intensity of one colour means changing at least two factors. If the rest of the spectrum is kept constant, the total light intensity changes as well as other colours as a fraction of the total intensity. And if the total light intensity is kept constant, an increase in one colour will at the same time lead to a decrease in the other colours. Such effects are very difficult to disentangle; the best way would be to apply a range of treatments where the spectrum is changed in different ways.

*Effects of daily light integral, PPFD, and photoperiod are often entangled*

In some studies the effects of photoperiod are examined while maintaining the same PPFD, meaning that the effects are entangled with those of the daily light integral (DLI). It might seem a better option to keep the DLI constant, but then the PPFD differs. Hence, in this case the photoperiod effects are entangled with PPFD. The effects of photoperiod *per se* could be studied by extending the day using low intensity lighting; however, the predictive value of these treatments for growth under high PPFD could be debated.

*Far-red: adding or substituting?*

Effects of FR are often studied by adding it to PAR light, as FR it is assumed not to be photosynthetically active; however, it clearly is photosynthetically active when combined with PAR (Zhen and Bugbee, 2020). Adding FR increases the photon flux density (PFD; ∼300–800 nm, μmol m^–2^ s^–1^) and results can be (partly) caused by more photons instead of a specific response to FR. Hence it would be relevant to (also) study replacing PAR by FR. Studies should clearly indicate both PPFD and PFD.

*Foliar shading*

When shaded by foliage, both the PFD and R:FR ratio decline, and both factors affect plant functioning and can interact. Experiments on shade-avoidance responses would often benefit from more care being taken in how PFD and FR are varied.

Many studies investigating FR explain the observed effects in relation to shade-avoidance responses. It should be realized that shade avoidance in nature or in open field crops is about responses to FR fractions that are higher than in solar light, while in climate chambers the FR fraction is usually lower.

*Independent replicates*

Independent replication is essential for being able to make sound conclusions (Rogers *et al*., 2021). This can be a challenge as it involves larger facilities and/or repetitions over time, which increases costs and labour demand. When light is the factor of interest, plants from the same compartment under given light conditions cannot be considered as independent replicates: this would leave no possibility to test differences between light conditions against random differences between compartments. Treating plants from the same experimental unit as independent observations underestimates the random variance and increases the chance of finding statistically significant treatment effects where in reality the treatments do not differ.

## Supplementary Material

eraf315_Supplementary_Data

## Data Availability

Not applicable.

## Supplementary data

The following supplementary data are available at *JXB* online.

Table S1. Overview of different photoreceptor mutants and transgenic lines in tomato.

Fig. S1. Relative yield of tomato cultivars as a function of year of cultivar release.

## Abbreviations

DM: dry mass
FKF1: FLAVIN BINDING, KELCH REPEAT, F-BOX1
FR: far-red
*g*_s_: stomatal conductance
*K*_leaf_: hydraulic conductance of leaves
LED: light-emitting diode
LKP2: LOV KELCH PROTEIN2
LOV: light, oxygen, voltage domain
PAR: photosynthetically active radiation
PFD: photon flux density
PPFD: photosynthetic PFD
SAS: shade-avoidance syndrome
