# Supplementary material for: Tomato in the spotlight: light regulation of whole-plant physiology
Source: J Exp Bot. 2025 Jul 15;76(21):6289–310. doi: 10.1093/jxb/eraf315 (PMC12646156; doi:10.1093/jxb/eraf315)
Supplement: eraf315_Supplementary_Data [file eraf315_supplementary_data.zip › eraf315 NEW -Supplementary data.pdf]

Tomato in the spotlight: Light regulation of whole-plant physiology in tomato

Ep Heuvelink[1], Liana G. Acevedo-Siaca[1], Bram Van de Poel[2,3], Laura Van der Jeucht[2,3], Silvere Vialet-Chabrand[1], Kathy Steppe[4], Yongran Ji[1], Oliver Körner[5], Paul Kusuma[1], Silvia Langer[1], Tao Li[6], Wim Van Ieperen[1], Julian C. Verdonk[1], Ana Cristina Zepeda[1], Yuqi Zhang[6], Leo F.M. Marcelis[1*]

Supplementary Data

Supplementary Table S1: Overview of the different photoreceptor mutants and transgenic lines in tomato (MM = Moneymaker; AC=Ailsa Craig).

**Photo-receptor**	**Anno-tation**	**Gene**	**Back-ground**	**Muta-gen**	**Mutation** (*nucleotide*/ amino acid)	**Phenotypes**	**Reference**
Phytochrome A	*fri*[1]	Solyc10g044670	MM	EMS	*A2327T*	insensitive to FR, longer roots	van Tuinen *et al.*, 1995a
Phytochrome A	*fri*[2]	Solyc10g044670	MM	EMS	*A2327T*	insensitive to FR, longer roots	van Tuinen *et al.*, 1995a
Phytochrome B1	*tri*[1]	Solyc01g059870	Breeding line GT	EMS	*C274**	elongation, elongated internodes, reduced anthocyanin	van Tuinen *et al.*, 1995b
Phytochrome B1	*tri*[2]	Solyc01g059870	Breeding line GT	EMS	*G3731A*	elongation, elongated internodes, reduced anthocyanin	van Tuinen *et al.*, 1995b
Phytochrome B1	*tri*[3]	Solyc01g059870	Breeding line GT	EMS	*G712T*	elongation, elongated internodes, reduced anthocyanin	van Tuinen *et al.*, 1995b
Phytochrome B1	*tri*[4]	Solyc01g059870	Breeding line GT	EMS	*C3567T*	elongation, elongated internodes, reduced anthocyanin	van Tuinen *et al.*, 1995b
phytochrome B2	*phyB2*	Solyc05g053410	MM	γ radiation	loss-of-function	wildtype	Weller *et al.*, 2000
phytochrome A, B1	*fri*[1]*,tri*[1]	Solyc10g044670, Solyc01g059870	MM and GT	cross	loss-of-function	elongation, reduced anthocyanin	Kerckhoffs *et al.*, 1997
phytochrome A, B1, B2	*phyAB1B2*	Solyc10g044670, Solyc01g059870, Solyc05g053410	MM and GT	cross, γ radiation	loss-of-function	elongation, reduced anthocyanin	Kerckhoffs *et al.*, 1999
phyochrome B1, B2	*phyB1B2*	Solyc01g059870, Solyc05g053410	MM and GT	cross	loss-of-function	elongation, reduced chlorophyll	Weller *et al.*, 2000
phytochrome F	*phyF*	Solyc07g045480	MM	CRISPR-Cas9	loss-of-function	elongation, longer roots	Balderrama *et al.*, 2023
pytochrome A, F	*phyAF*	Solyc10g044670, Solyc07g045480	MM	cross	loss-of-function	longer roots	Balderrama *et al.*, 2023
phytochrome B1, F	*phyB1F*	Solyc01g059870, Solyc07g045480	MM	cross	loss-of-function	elongation	Balderrama *et al.*, 2023
phytochrome B1, B2, F	*phyB1B2F*	Solyc01g059870, Solyc05g053410, Solyc07g045480	MM	cross	loss-of-function		Balderrama *et al.*, 2023

phytochrome E	*phyE*	Solyc02g071260	MM and GT	amiRNA	50% loss-of-function	wildtype	Schrager-Lavelle *et al.*, 2016
phytochrome B1, E	*phyB1E*	Solyc01g059870, Solyc02g071260	MM and GT	cross	loss-of-function		Schrager-Lavelle *et al.*, 2016
phytochrome B2, E	*phyB2E*	Solyc05g053410, Solyc02g071260	MM and GT	cross	loss-of-function		Schrager-Lavelle *et al.*, 2016
phytochrome B1, B2, E	*phyB1B2E*	Solyc01g059870, Solyc05g053410, Solyc02g071260	MM and GT	cross	loss-of-function		Schrager-Lavelle *et al.*, 2016
phytochrome A, B1, B2, E	*pyhAB1B2E*	Solyc10g044670, Solyc01g059870, Solyc05g053410, Solyc02g071260	MM and GT	cross	loss-of-function		Schrager-Lavelle *et al.*, 2016
phytochrome chromophore	*au*		First Early	X radiation and ^{32}P treatment	loss-of-function	reduced chlorophyll and anthocyanin, elongation	Lesley & Lesley, 1956
phytochrome chromophore	*au*		AC	cross	loss-of-function	reduced chlorophyll and anthocyanin, elongation	Darby *et al.*, 1978
phytochrome chromophore	au^{w}		MM	cross	loss-of-function	reduced chlorophyll and anthocyanin, elongation	Koornneef *et al.*, 1981 & 1985
phytochrome chromophore	*yg-2*	Solyc12g009470	unknown	EMS	loss-of-function	reduced chlorophyll and anthocyanin, elongation	Burdick, 1958
phytochrome chromophore	$yg\text{-}2^{aud}$	Solyc12g009470	AC	unknown	loss-of-function	reduced chlorophyll and anthocyanin, elongation	van Tuinen *et al.*, 1996
phytochrome chromophore	$yg\text{-}6/au^{6}$		unknown	EMS	loss-of-function	reduced chlorophyll and anthocyanin, elongation	Burdick, 1960
phytochrome chromophore, phytochrome B1	au,tri^{1}	Solyc01g059870	MM and GT	cross	loss-of-function	late accumulation of anthocyanin	Kerckhoffs *et al.*, 1997
phytochrome chromophore	$au^{w},yg\text{-}2^{aud}$	Solyc12g009470	MM and AC	cross	loss-of-function	reduced chlorophyll	van Tuinen *et al.*, 1996
cryptochrome 1a	*cry1-1*	Solyc04g074180	AC	EMS	*G1680A*	elongation, larger seeds, longer roots, increased chlorophyll, increased total leaf area	Weller *et al.*, 2001
cryptochrome 1a	*cry1-2*	Solyc04g074180	MM	γ radiation	*A192-*	elongation, curled hypocotyl, reduced chlorophyll in fruits	Weller *et al.*, 2001
phytochrome A, cryptochrome 1a	*phyA cry1*	Solyc10g044670, Solyc04g074180	MM	cross	loss-of-function		Weller *et al.*, 2001
phytochrome B1, cryptochrome 1a	*phyB1 cry1*	Solyc01g059870, Solyc04g074180	MM	cross	loss-of-function		Weller *et al.*, 2001
phytochrome A, B1, cryptochrome 1a	*phyA phyB1 cry1*	Solyc10g044670, Solyc01g059870, Solyc04g074180	MM	cross	loss-of-function	reduced chlorophyll and anthocyanin, elongation	Weller *et al.*, 2001
phytochrome A, B1, B2, cryptochrome 1a	*phyA phyB1 phyB2 cry1*	Solyc10g044670, Solyc01g059870, Solyc05g053410,	MM	cross	loss-of-function	lethal	Weller *et al.*, 2001

		Solyc04g074180					
cryptochrome 1a	*OE-CRY1a-1*	Solyc04g074180	MM	35S	overexpression	short, decreased total leaf area, decreased chlorophyll in leaves, increased chlorophyll in fruits, small fruits	Liu *et al.*, 2018
cryptochrome 1a	*OE-CRY1a-2*	Solyc04g074180	MM	35S	overexpression	short, decreased total leaf area, decreased chlorophyll in leaves, increased chlorophyll in fruits, small fruits	Liu *et al.*, 2018
cryptochrome 2	*CRY2-silenced*	Solyc09g090100	MM	VIGS	loss-of-function	slight internode elongation	Giliberto *et al.*, 2005
cryptochrome 2	*CRY2-OX*	Solyc09g090100	MM	35S	overexpression	short, increased chlorophyll and anthocyanin levels in leaves, delayed flowering, decreased apical dominance	Giliberto *et al.*, 2005
cryptochrome 2	*cry2-1*	Solyc09g090100	MM	EMS	W318*	elongation	Fantini *et al.*, 2019
cryptochrome 1a, 2	*cry1a/cry2*	Solyc04g074180, Solyc09g090100	MM	cross	loss-of-function	larger seeds, elongation, longer roots, reduced chlorophyll in fruits	Fantini *et al.*, 2019
phototropin1	*Nps1*	Solyc11g072710	AC	EMS	*G1484A*	delayed/sluggish deetiolation, leaf curling, epinasty, more biomass, reduced carotenoid levels in fruits	Sharma *et al.*, 2014
phototropin1	*Nps1*	Solyc11g072710	AC	CRISPR-Cas9	loss-of-function		Kilambi *et al.*, 2021
UVR8	*UVR8Ri*	Solyc05g018615	AC	RNAi	silencing	wildtype, hypersensitive to UV-B	Li *et al.*, 2018
UVR8	*UVR8OE*	Solyc05g018615	AC	35S	overexpression	wildtype, hardly sensitive to UV-B	Li *et al.*, 2018
UVR8	*uvr8*	Solyc05g018615	AC	CRISPR-Cas9	loss-of-function	wildtype, insensitive to UV-B	Liu *et al.*, 2020

References

Balderrama D, Barnwell S, Carlson KD, Salido E, Guevara R, Nguyen C, Madlung A. 2023. Phytochrome F mediates red light responsiveness additively with phytochromes B1 and B2 in tomato. Plant Physiology **191**, 2353-2366.

Burdick AB. 1958. New mutants. Tomato Genetics Cooperative Report **8**, 8-9.

Burdick AB. 1960. New gene symbols. Tomato Genetics Cooperative Report **10**, 8-9.

Darby LA. 1978. Isogenic lines of tomato fruit colour mutants.

Fantini E, Sulli M, Zhang L, Aprea G, Jiménez-Gómez JM, Bendahmane A, Gaetano P, Giuliano G, Facella P. 2019. Pivotal roles of cryptochromes 1a and 2 in tomato development and physiology. Plant Physiology **179**, 732-748.

Giliberto L, Perrotta G, Pallara P, Weller JL, Fraser PD, Bramley PM, Fiore A, Tavazza M, Giuliano, G. 2005. Manipulation of the blue light photoreceptor cryptochrome 2 in tomato affects vegetative development, flowering time, and fruit antioxidant content. Plant Physiology **137**, 199-208.

Lesley JW, Lesley MM. 1956. Effect of seed treatments with X-ray and phosphorus 32 on tomato plants of first, second, and third generations. Genetics **41**, 575.

Li H, Li Y, Deng H, *et al*. 2018. Tomato UV-B receptor SlUVR8 mediates plant acclimation to UV-B radiation and enhances fruit chloroplast development via regulating SlGLK2. Scientific Reports **8**, 6097.

Liu C, Ahammed GJ, Wang G, Xu C, Chen K, Zhou Y, Yu, JQ. 2018. Tomato CRY1a plays a critical role in the regulation of phytohormone homeostasis, plant development, and carotenoid metabolism in fruits. Plant, Cell & Environment **41**, 354-366.

Liu X, Zhang Q, Yang G, Zhang C, Dong H, Liu Y, Yin R, Lin L. 2020. Pivotal roles of Tomato photoreceptor SlUVR8 in seedling development and UV-B stress tolerance. Biochemical and Biophysical Research Communications **522**, 177-183.

Kerckhoffs LHJ, Kelmenson PM, Schreuder MEL, Kendrick CI, Kendrick RE, Hanhart CJ, Koornneef M, Cordonnier-Pratt MM. 1999. Characterization of the gene encoding the apoprotein of phytochrome B2 in tomato, and identification of molecular lesions in two mutant alleles. Molecular and General Genetics MGG **261**, 901-907.

Kerckhoffs LHJ, Sengers MMT, Kendrick RE. 1997. Growth analysis of wild-type and photomorphogenic-mutant tomato plants. Physiologia Plantarum **99**, 309-315.

Kilambi HV, Dindu A, Sharma K, *et al.* 2021. The new kid on the block: a dominant-negative mutation of phototropin1 enhances carotenoid content in tomato fruits. The Plant Journal **106**, 844-861.

Koornneef M, Cone JW, Dekens RG, O'Herne-Roberts EG, Spruit CJP, Kendrick RE. (1985). Long hypocotyl, phytochrome, mutants in tomato. In European Symposium on Photomorphogenesis, Wageningen **72.**

Koornneef M, van der Veen JH, Spruit CJP, Karssen CM. 1981. The isolation and use of mutants with an altered germination behaviour in Arabidopsis thaliana and tomato. In Induced mutations-Tool for plant improvement, **227-232.**

Schrager-Lavelle A, Herrera LA, Maloof JN. 2016. Tomato phyE is required for shade avoidance in the absence of phyB1 and phyB2. Frontiers in Plant Science **7**, 1275.

Sharma S, Kharshiing E, Srinivas A, *et al.* 2014. A dominant mutation in the light-oxygen and voltage2 domain vicinity impairs phototropin1 signaling in tomato. Plant Physiology **164**, 2030-2044.

Van Tuinen A, Hanhart CJ, Kerckhoffs LHJ, Nagatani A, Boylan MT, Quail PH, Kendrick RE, Koornneef M. 1996. Analysis of phytochrome-deficient yellow-green-2 and aurea mutants of tomato. The Plant Journal **9**, 173-182.

Van Tuinen A, Kerckhoffs LHJ, Nagatani A, Kendrick RE, Koornneef M. 1995a. Far-red light-insensitive, phytochrome A-deficient mutants of tomato. Molecular and General Genetics MGG **246**, 133-141.

Van Tuinen A, Kerckhoffs, LHJ, Nagatani A, Kendrick RE, Koornneef M. 1995b. A temporarily red light-insensitive mutant of tomato lacks a light-stable, B-like phytochrome. Plant Physiology **108**, 939-947.

Weller JL, Perrotta G, Schreuder ME, Van Tuinen A, Koornneef M, Giuliano G, Kendrick RE. 2001. Genetic dissection of blue-light sensing in tomato using mutants deficient in cryptochrome 1 and phytochromes A, B1 and B2. The Plant Journal **25** 427-440.

Weller JL, Schreuder ME, Smith H, Koornneef M, Kendrick RE. 2000. Physiological interactions of phytochromes A, B1 and B2 in the control of development in tomato. The Plant Journal **24**, 345-356.

Supplementary Fig. S1

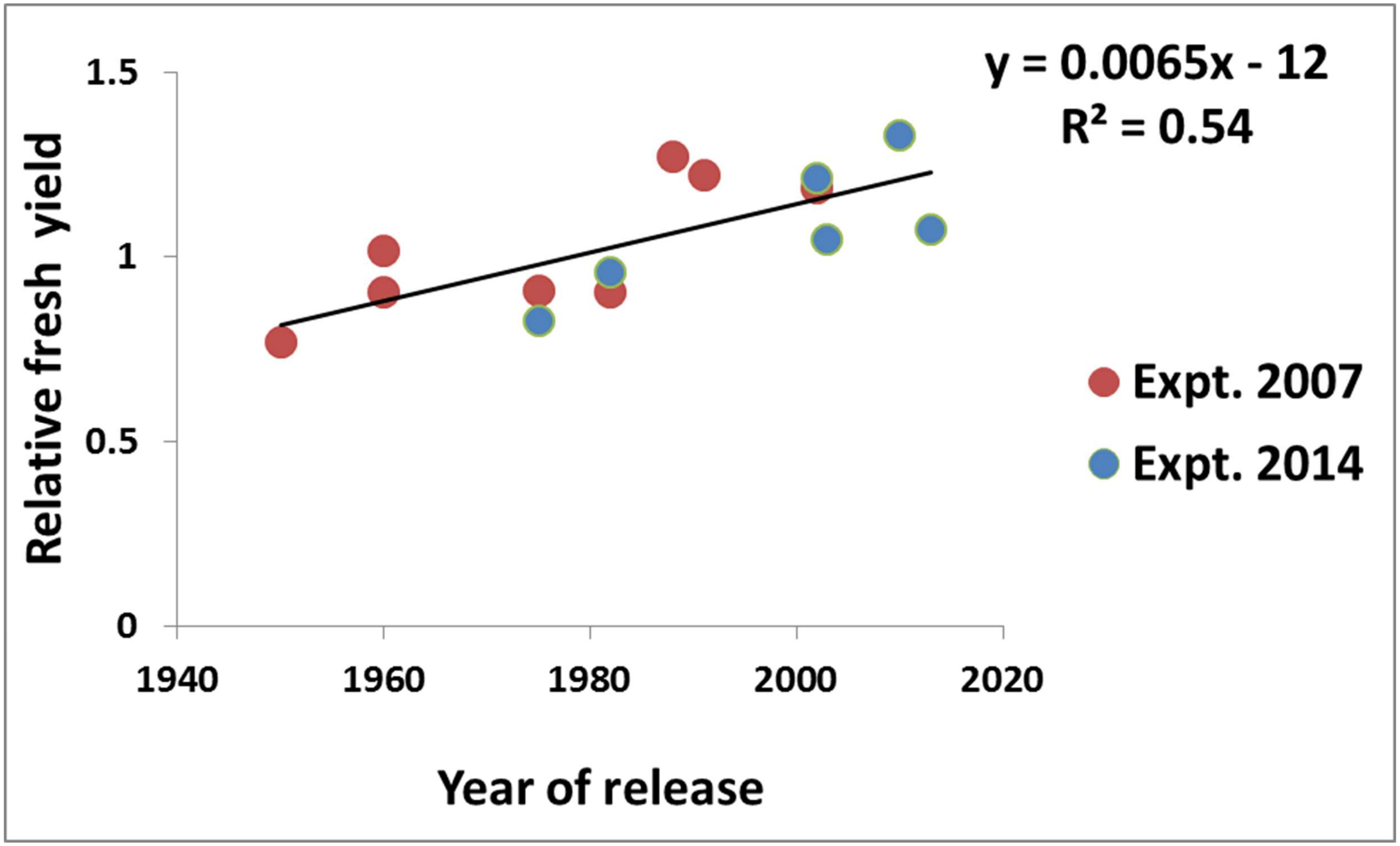

Fig. S1. Relative yield of tomato cultivars as a function of year of cultivar release determined in two greenhouse experiments: Expt. 2007 was conducted from August to November 2007 using eight cultivars released between 1950 and 2002 (published in Higashide et al., 2009) and Expt. 2014 was conducted from June to December 2014 using six cultivars released between 1975 and 2013. Three cultivars were the same in both experiments and within an experiment yields were expressed relative to the average yield of these 3 cultivars. As Expt. 2014 had a longer cultivation period than Expt. 2007, relative yield rather than absolute yield had to be used for combining both experiments.
