# Supplementary material for: Tomato in the spotlight: light regulation of whole-plant physiology
Source: J Exp Bot. 2025 Jul 15;76(21):6289–310. doi: 10.1093/jxb/eraf315 (PMC12646156; doi:10.1093/jxb/eraf315)
Supplement: eraf315_Supplementary_Data [file eraf315_supplementary_data.zip › JXB_Supplementary_Figure_S1.docx]

**Tomato in the spotlight: Light regulation of whole-plant physiology in tomato**

Ep Heuvelink^1^, Liana G. Acevedo-Siaca^1^, Bram Van de Poel^2,3^, Laura Van der Jeucht^2,3^, Silvere Vialet-Chabrand^1^, Kathy Steppe^4^, Yongran Ji^1^, Oliver Körner^5^, Paul Kusuma^1^, Silvia Langer^1^, Tao Li^6^, Wim Van Ieperen^1^, Julian C. Verdonk^1^, Ana Cristina Zepeda^1^, Yuqi Zhang^6^, Leo F.M. Marcelis^1*^

Supplementary Data

**Supplementary Figure S1**


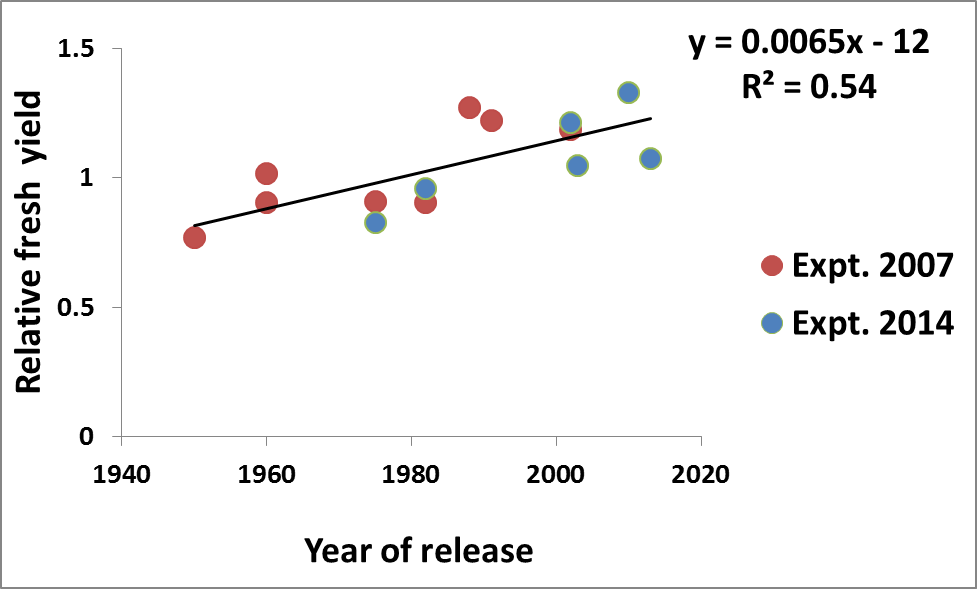


Figure S1. Relative yield of tomato cultivars as a function of year of cultivar release determined in two greenhouse experiments: Expt. 2007 was conducted from August to November 2007 using eight cultivars released between 1950 and 2002 (published in Higashide et al., 2009) and Expt. 2014 was conducted from June to December 2014 using six cultivars released between 1975 and 2013. Three cultivars were the same in both experiments and within an experiment yields were expressed relative to the average yield of these 3 cultivars. As Expt. 2014 had a longer cultivation period than Expt. 2007, relative yield rather than absolute yield had to be used for combining both experiments.
